# Evaluation of the conversion rate as it relates to preoperative risk factors and surgeon experience: a retrospective study of 4013 patients undergoing elective laparoscopic cholecystectomy

**DOI:** 10.1186/s12893-021-01152-z

**Published:** 2021-03-20

**Authors:** Szabolcs Ábrahám, Tibor Németh, Ria Benkő, Mária Matuz, Dániel Váczi, Illés Tóth, Aurél Ottlakán, László Andrási, János Tajti., Viktor Kovács, József Pieler, László Libor, Attila Paszt, Zsolt Simonka, György Lázár

**Affiliations:** 1 Department of Surgery, Szent-Györgyi Albert Medical and Pharmaceutical Center, University of Szeged, Semmelweis u. 8, 6725 Szeged, Hungary; 2 Department of Clinical Pharmacy, University of Szeged, Szeged, Hungary

**Keywords:** Elective, Laparoscopic cholecystectomy, Conversion rate, Risk factors, Predictive factors, Surgical experience

## Abstract

**Background:**

Our aim is to determine the relationships among patient demographics, patient history, surgical experience, and conversion rate (CR) during elective laparoscopic cholecystectomies (LCs).

**Methods:**

We analyzed data from patients who underwent LC surgery between 2005 and 2014 based on patient charts and electronic documentation. CR (%) was evaluated in 4013 patients who underwent elective LC surgery. The relationships between certain predictive factors (patient demographics, endoscopic retrograde cholangiopancreatography (ERCP), acute cholecystitis (AC), abdominal surgery in the patient history, as well as surgical experience) and CR were examined by univariate analysis and logistic regression.

**Results:**

In our sample (N = 4013), the CR was 4.2%. The CR was twice as frequent among males than among females (6.8 vs. 3.2%, *p* < 0.001), and the chance of conversion increased from 3.4 to 5.9% in patients older than 65 years. The detected CR was 8.8% in a group of patients who underwent previous ERCP (8.8 vs. 3.5%, *p* < 0.001). From the ERCP indications, most often, conversion was performed because of severe biliary tract obstruction (CR: 9.3%). LC had to be converted to open surgery after upper and lower abdominal surgeries in 18.8 and 4.8% cases, respectively. Both AC and ERCP in the patient history raised the CR (12.3%, *p* < 0.001 and 8.8%, *p* < 0.001). More surgical experience and high surgery volume were not associated with a lower CR prevalence.

**Conclusions:**

Patient demographics (male gender and age > 65 years), previous ERCP, and upper abdominal surgery or history of AC affected the likelihood of conversion. More surgical experience and high surgery volume were not associated with a lower CR prevalence.

## Background

In all, 10–15% of developed societies are affected by cholecystolithiasis [1]. The number of cholecystectomies that are performed has gradually increased worldwide since 1950. With the introduction of the operative technique for laparoscopic cholecystectomy (LC) and the popularity of minimally invasive surgery, cholecystectomies became a routine procedure in the 1990s. [1, 2]. At our institution the first LC was performed in March, 1991 (Béla Baltás). Currently, 90% of acute and elective cholecystectomies are performed laparoscopically [3–5], and the portion of open surgeries is decreasing. The conversion rate (CR) could be a quality indicator of surgical practice in the case of laparoscopic surgeries. The CR shows the portion of converted cholecystectomies compared with all gallbladder removal surgeries that begin as laparoscopic procedures. During acute and elective LC surgeries of 178,875 patients, the national CR was 4.86% in Hungary between 2005 and 2013, and this value was based on the itemized healthcare data of the National Healthcare Services Center (formerly National Institute of Quality and Organizational Development in Healthcare and Medicines) (OENO:55118; OENO:55119) [6, 7].

According to a recently published systematic review article, the average CR varies widely between 1 and 30% [8–11]. There are preoperative and intraoperative indications for conversion. In addition to the general patient characteristics such as male gender [12], older age, obesity, the presence of other concomitant illnesses, and worse physical status of the patient (e.g., higher American Society of Anesthesiologist (ASA) score) have an effect on laparoscopic technique during cholecystectomies. The gallbladder and biliary tract status, as confirmed by abdominal ultrasound, and other diseases (such as biliary tract obstruction and acute biliary pancreatitis), which indicate the necessity of endoscopic retrograde cholangiopancreatography (ERCP) before surgery, also have a significant role in conversion [8, 9, 13–17]. Besides the abovementioned factors, surgical proficiency and competence can also affect the likelihood of conversion [13].

Our research was motivated by the fact that few articles have been published on elective cholecystectomies in terms of the conversion rate [18–20], hence there is research gap. Since conversion can give rise to several negative consequences such as longer surgery time, prolonged hospitalization, slower recovery [8], higher rates of readmission, and increasing morbidity and mortality [9], it is essential to assess the potential risk factors for conversion.

Our aim is to determine the relationship among patient demographics, patient history, surgical experience, and the conversion rate (CR) during elective LCs.

## Methods

Ethical permission for this study was obtained from the Regional Human Biomedical Research Ethics Committee of the University of Szeged (74/2016-SZTE).

We retrospectively analyzed cholecystectomies performed between 2005 and 2014 in the Department of Surgery, University of Szeged. Altogether, cholecystectomy was performed in 4,438 patients over the study period. The indications for elective cholecystectomies were symptomatic cholecystolithiasis and conditions with previous biliary tract obstruction, acute biliary pancreatitis, and acute cholecystitis. Patients were allocated to surgeons independently from the expected difficulty of the cholecystectomy.

**Fig. 1:**
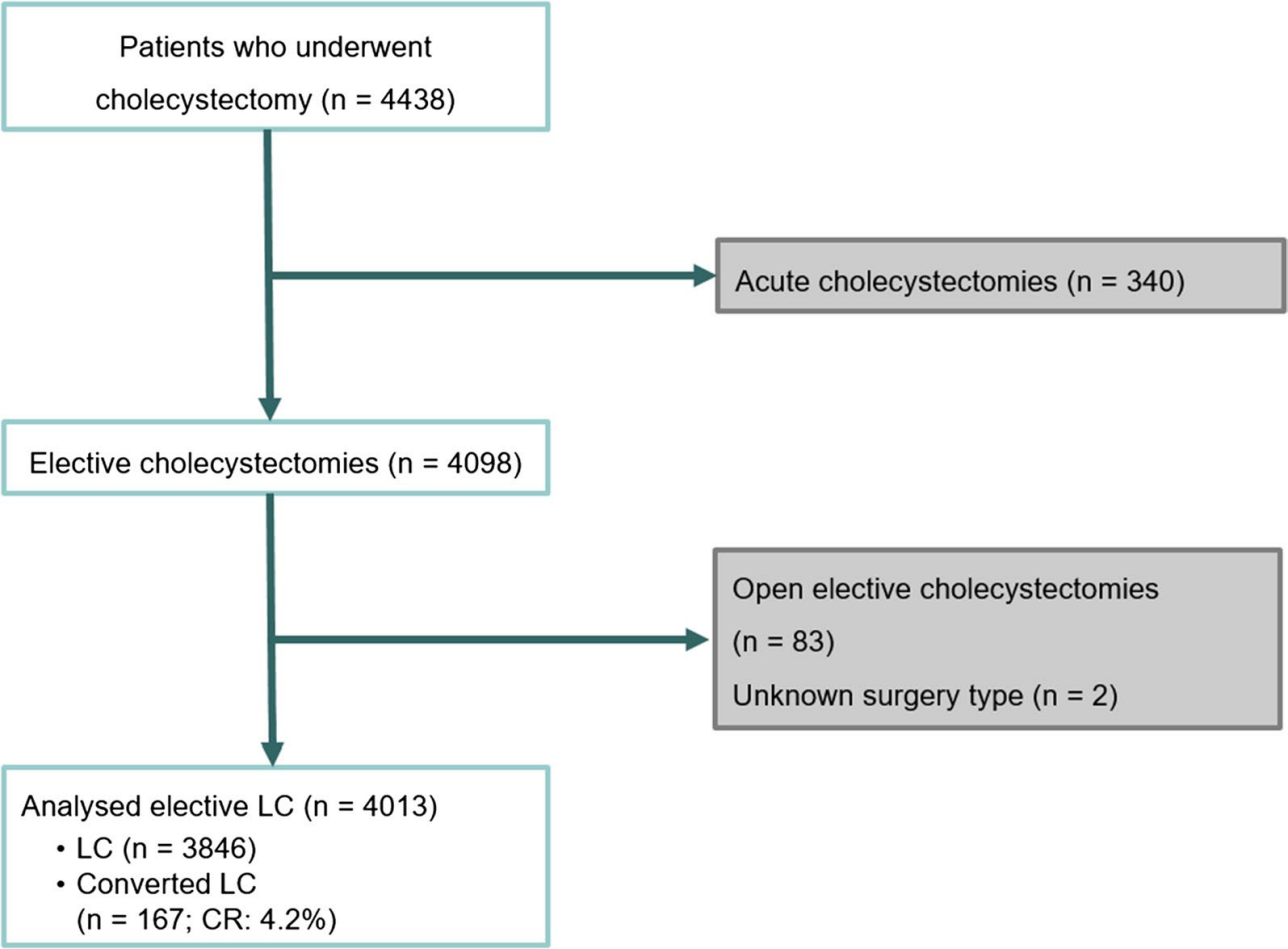
Flowchart showing the number of patients who met the inclusion criteria, those who were excluded, and those who were enrolled in the final analysis (*LC* laparoscopic cholecystectomy, *CR* conversion rate)

Patients who underwent urgent/early cholecystectomies (acute cholecystectomies) due to acute cholecystitis were excluded from the study. Acute cholecystitis were defined by Tokyo Guideline 2018 [21]. We defined the surgery as elective cholecystectomy after acute cholecystitis, if at least 3 weeks elapsed since the hospital admission due to acute cholecystitis. Primary open elective cholecystectomies were also excluded from the analysis (see Fig. 1). Decision on the exclusion was done by two general surgeons and discrepancies were resolved by dialogue. The CR was determined as a percentage and was based on the ratio of the overall converted surgeries and the sum of converted and laparoscopic surgeries (number of converted surgeries/(number of converted surgeries + number of LC surgeries) × 100).

The CR was stratified by gender and patient age (18–65 years and 65 + years). We examined the impact of previous upper and lower abdominal surgeries and that of preoperative ERCP due to different indications on the conversion rate. The indications for preoperative ERCP were divided into four categories: moderate biliary tract obstruction (elevated serum alkaline phosphatase and gamma-glutamyl transferase, normal bilirubin level, and biliary tracts without dilatation), severe biliary tract obstruction (elevated serum bilirubin and ultrasound findings of biliary tract dilatation), acute biliary pancreatitis, and other indications. Moreover, we examined how the CR was affected by acute cholecystitis and related ultrasound-guided transhepatic drainage prior to gallbladder removal surgery and by the time elapsed between acute cholecystitis and elective cholecystectomy. We also analyzed the impact of surgical proficiency and surgeon experience on the CR. In our department, every surgeon perform LC irrespective of sub-specialisation. Each surgeon was categorized into three groups according to annual surgery volume for cholecystectomy: low-volume surgeons (≤ 10 surgeries per year), moderately high-volume surgeons (11–40 surgeries per year), and high-volume surgeons (> 40 surgeries per year). We created three additional groups among the surgeons based on their clinical experience at the time of the surgery, calculated in years (≤ 6 years of experience, 7–15 years of experience, and > 15 years of experience). In Hungary, general surgery residency training requires six years. Surgical residents operate under supervision before specialization, at which point they perform surgeries on their own. In the study period, the surgical method was standard and technical conditions has not changed substantially.

The potential influencing factors for conversion were examined by univariate analysis (Welch’s two-sample t-test and Fisher’s exact test) and by logistic regression.

## Results

According to the aforementioned exclusion criteria, the data of 4013 patients who underwent elective cholecystectomy during the study period were analyzed (Table 1). LC was performed in 3846 cases (95.8%), and LC was converted to open surgery in 167 patients (4.2%) (Fig. 1). The CR was twice as high among males (6.8% vs. 3.2% in females), and patient’s age was also higher in the converted group (Table 1). The conversion rate was minimally elevated after lower abdominal surgeries (4.8%), but reached 18.8% after upper abdominal surgeries. Both acute cholecystitis and ERCP in the patient history raised the CR (12.3%, *p* < 0.001 and 8.8%, *p* < 0.001; see Table 1).

**Table 1:** Impact of patient/physician-related characteristics on conversion calculated using univariate analysis

				Laparoscopic cholecystectomy	Converted cholecystectomy	*P* value
Number of patients (%)				3846 (95.84%)	167 (4.16%)	–
Age (years)	Mean ± SD			54.09 ± 14.72	61.54 ± 13.60	< 0.001^1^
	20–65			2893	101 (3.37%)	< 0.001^2^
	65 +			953	66 (6.48%)	
	80 +			86	15 (14.85%)	–
Gender	Men			1009	74 (6.83%)	< 0.001^2^
	Women			2837	93 (3.17%)	
Lower abdominal surgery (N/A: 397)	No			2208	84 (4.76%)	0.116^2^
	Yes			1261	63 (3.66%)	
Upper abdominal surgery (N/A:397)	No			3335	116 (3.36%)	< 0.001^2^
	Yes			134	31 (18.78%)	
Acute cholecystitis in patient history (N/A:350)	No			3258	114 (3.38%)	< 0.001^2^
	Yes			256	35 (12.03%)	
	Timing of surgery (N/A: 41)		Between 3–6 weeks	42	6 (12.50%)	0.8044
			After 6 weeks	179	23 (11.39%)	
ERCP in patient history (N/A:1)	No			3411	125 (3.54%)	< 0.001^2^
	Yes			434	42 (8.82%)	
	Indication for ERCP	Acute biliary pancreatitis		98	9	Not tested
		Moderate biliary tract obstruction		90	9	
		Severe biliary tract obstruction		184	20	
		Other indication		62	4	
Surgeon’s annual surgery volume (N/A:69)	1–10 per year			889	38 (4.10%)	0.685^3^
	11–40 per year			2191	102 (4.45%)	
	> 40 per year			697	27 (3.73%)	
	Mean ± SD			27.53 ± 22.45	2 7.26 ± 20.88	0.881^1^
Surgical experience (years)	0–6 years			699	20 (2.78%)	0.003^3^
	7–15 years			1209	42 (3.36%)	
	> 15 years			1869	105 (5.32%)	
	Mean ± SD			16.23 ± 9.46	21.09 ± 11.99	< 0.001^1^

^1^Welch's t-test; ^2^Fisher exact test; ^3^Chi-Square test

*N* number of patients, *ERCP* endoscopic retrograde cholangiopancreatography

The indication of preoperative ERCP did not show any correlation with the CR (Table 1). Overall, of 256 patients with a history of acute cholecystitis, 34 required conversion to an open procedure during the elective cholecystectomy (after achievement of the non-inflammatory stage).

The different timing (between 3–6 weeks or after 6 weeks) of elective cholecystectomy after acute inflammation has not been associated with conversion rate (Table 1.).

During the 10-year study period, 56 surgeons performed the abovementioned 4013 surgeries. The CR ranged between 3.7% and 4.4% in the different groups of surgeons (low-volume, moderately high-volume, or high-volume surgeons). An analysis of the time that elapsed since graduation (surgeon’s experience) found that the CR was inversely related to surgical experience: (Table 1). The assessed patient characteristics and risk factors were similar in the three surgeons’ group.

According to the univariate analyses, older age, male gender, previous upper abdominal surgery, acute cholecystitis, ultrasound-guided drainage, and preoperative ERCP were more frequent in the converted group (Table 1). All of these factors confirmed a higher likelihood of conversion (odds ratio above 1) based on the results of the multivariate logistic regression (Table 2): age (OR: 1.032; CI: 1.019–1.045), male gender (OR: 1.582; CI: 1.104–2.268), ultrasound-guided drainage (OR: 2.218; CI: 0.788–6.245), preoperative ERCP (OR: 2.190; CI: 1.441–3.329), previous upper abdominal surgery (OR: 5.551; CI: 3.458–8.750), and previous acute cholecystitis (OR: 3.419; CI: 2.219–5.268). The most clinically relevant factors with the highest odds ratios were previous upper abdominal surgery and acute cholecystitis (Table 2).

**Table 2:** Impact of patient/physician-related characteristics on conversion analyzed using multivariate analysis (logistic regression)

	B	p	OR	95% CI for OR	
				Lower	Upper
Gender (male)	0.459	0.013	1.582	1.104	2.268
Age	0.031	< 0.001	1.032	1.019	1.045
Previous acute cholecystitis	1.229	< 0.001	3.419	2.219	5.268
Previous US-guided gallbladder drainage	0.796	0.132	2.218	0.788	6.245
Previous upper abdominal surgery	1.705	< 0.001	5.501	3.458	8.750
Previous lower abdominal surgery	0.269	0.138	1.308	0.918	1.866
Previous ERCP	0.784	< 0.001	2.190	1.441	3.329
Time since graduation (baseline: 0–6 years)					
Time since graduation: 7–15 years	0.211	0.499	1.235	0.670	2.274
Time since graduation: > 15 years	0.552	0.051	1.737	0.997	3.026
Constant	− 6.099	< 0.001			

*B* regression coefficient, *CI* confidence interval, *OR* odds ratio, *CI* confidence interval, *ERCP* endoscopic retrograde cholangiopancreatography, *US* ultrasound

## Discussion

Laparoscopic cholecystectomy (LC) is the “gold standard” for the surgical management of symptomatic cholecystolithiasis. Compared with open cholecystectomy, the minimally invasive laparoscopic technique causes minor surgical stress and less postoperative pain for patients, and it is associated with shorter hospitalization, quicker recovery, better cosmetic results, and lower prevalence of impaired wound healing. Thus, it can be considered a more economical solution than open surgery [13, 22].

Most publications on the CR do not distinguish between emergency and elective surgeries [8–10, 12, 22], hence the novelty of this work that it focus on elective surgeries. Elective and emergency surgeries are performed for different indications. In addition, the patient’s condition and the intraoperative circumstances may result in different conversion rates. We focused our research solely on elective surgeries. In these surgeries, we examined patient- and surgeon-related characteristics that may influence the CR.

In our study, the CR was found to be 4.2% during elective LC surgeries. Prior clinical studies also drew attention to risk factors for conversion. In the retrospective study by van der Steeg et al. [13], which included 972 patients and focused on both acute and elective surgeries, male gender, age older than 65 years, acute cholecystitis, and obstructive icterus were identified as the risk factors for the conversion of LC. Ercan et al. [20] also analyzed the predictive factors of conversion. According to their findings using multivariate analysis, previous abdominal surgery, preoperative ERCP, high-grade adhesions, and scleroatrophic gallbladder were the predictive factors for conversion. In the systematic review and meta-analysis that included 32 studies published by Rothman et al. in 2016 [9], the preoperative risk factors for conversion were investigated in 460,995 patients. They found that in addition to the echo-confirmed gallbladder status (gallbladder wall thickening greater than 4–5 mm and contracted gallbladder), age older than 60–65 years, male gender, and existing acute cholecystitis were the risk factors for the conversion of LC to open surgery. However, they did not confirm any correlation between previous abdominal surgeries and conversion to open surgery. This latter result is partly inconsistent with our findings, in which previous lower abdominal surgery did not have a considerable influence on conversion, but the likelihood of conversion significantly increased after upper abdominal surgery. In their prospective study involving 8820 patients, Sutcliffe et al. [8] found the following six significant predictive factors: older age, male gender, indication for surgery, ASA score, thick-walled gallbladder, and common bile duct dilatation.

Although the likelihood of conversion was not associated with the surgeon’s annual surgery volume, surgical proficiency discreetly influenced the CR according to this study. Our study indicates that surgical proficiency, that is, the time spent in clinical practice, surprisingly does not lead to decreased conversion rates and that it was associated with a higher CR (Table 1). Surgical residents had to convert LC surgeries less often than more experienced surgeons (2.8 vs. 3.4% and 2.8 vs. 5.3%). In 2015, Rothman et al. [23] conducted a prospective cohort study consisting of 36,231 patients. They did not examine the surgeon’s annual surgical volume in relation to conversion, but they did analyze the total number of surgeries performed before the study period. They compared moderately high-volume surgeons (50 to 100 surgeries) and surgeons with more than 200 LC surgeries. Conversion was almost twice as high in the latter group (OR: 1.80; 95% CI: 1.51–2.14). Thus, it seems that a lower volume of surgeries is not a risk factor for conversion [24, 25]. In a study consisting of 37,636 patients, Jolley et al. [26] analyzed patients’ medical data and surgical complications that emerged during the learning curve of resident surgeons. That study came to a similar conclusion as we did in terms of CRs of young resident surgeons: resident involvement did not result in a higher number of conversions [26]. Further investigations are needed to explore the reasons for the lower-than-expected CR among surgeons with less experience, butas studied patient characteristics and risk factors for CR were similar among surgeon’s groups with different experience (0–6 years, 7–15 years, 15 + years) this might not affect results (we cannot say that younger surgeons operated less complicated patients). 

One obvious reason could be behavioural factors (trust and enhanced experience in open surgeries, opt for safe and predictable conversion instead of continuing LC with unpredictable surgery duration) resulted in higher CR in the experienced surgeons group.

The limitation of our research is that some preoperative conditions, patient’s risk factors (e.g. body mass index, ASA score, gallbladder and biliary tract status) or intraoperative factors (e.g. length of surgery) or duration of symptoms were not analyzed as predictive factors of CR. Due to the retrospective nature of data collection, certain type of bias (e.g. reporting bias) cannot be excluded, however its effect on study findings is considered minimal. Furthermore, this was a single-center study, which limits the generalizability of the results.

## Conclusion

In our study, in addition to patient demographics (male gender and age > 65 years) and previous ERCP, we found that history of acute cholecystitis and upper abdominal surgery were the most influential factors in conversion. Knowledge of these factors is important because we can predict the anticipated difficulties and the likelihood of conversion before surgery. Both the operating surgeon and the assistant can prepare for the surgery, and we can account for the possibility of incidental open surgery and the difficulties that may arise from prolonged anesthesia.

## Data Availability

This retrospective clinical study contains clinical data from the electronic medical record in the Department of Surgery, University of Szeged. Additional information is available from the corresponding author on reasonable request from the editor.

## Abbreviations

LC: Laparoscopic cholecystectomy
CR: Conversion rate
AC: Acute cholecystitis
ASA: American Society of Anesthesiologist
ERCP: Endoscopic retrograde cholangiopancreatography
OR: Odds ratio
B: Regression coefficient
CI: Confidence interval
CI: Confidence interval
US: Ultrasound
